# Targeting Undruggable Protein Interactions with DNA Aptamers: Inhibition of the Interaction Between *Yersinia* Outer Protein M and Human DEAD-Box Helicase 3

**DOI:** 10.3390/ijms27094038

**Published:** 2026-04-30

**Authors:** Oğuz Gök, Özge Uğurlu, Canan Özyurt, Serap Evran

**Affiliations:** 1 Department of Biochemistry, Faculty of Science, Ege University, 35100 Izmir, Türkiye; oguzgok34@gmail.com; 2 Department of Medical Services and Techniques, Hatay Vocational School of Health Services, Hatay Mustafa Kemal University, 31060 Hatay, Türkiye; ozge.ugurlu@mku.edu.tr; 3 Department of Chemistry and Chemical Processing Technologies, Lapseki Vocational School, Canakkale Onsekiz Mart University, 17800 Canakkale, Türkiye; cananozyurt@comu.edu.tr

**Keywords:** DNA aptamer, SELEX, protein–protein interaction, inhibitor, plague

## Abstract

The plague, caused by *Yersinia pestis*, has resulted in significant mortality over the past century. Despite advances in antimicrobial therapy, plague remains a re-emerging infectious disease with ongoing outbreaks and increasing concerns regarding antimicrobial resistance. Today, plague cases are still being reported, and the loss of effectiveness of treatment methods remains a major challenge. Therefore, effective treatment strategies are needed. In this study, we aimed to develop aptamers specific to *Yersinia* outer protein M (YopM), a key immunosuppressive protein that is essential for virulence. Our goal was to develop an aptamer that binds to YopM and inhibits its interaction with the human DEAD-box helicase 3 (DDX3) protein. YopM-DDX3 protein interaction was targeted because of its key role in nucleocytoplasmic shuttling of YopM. To achieve this, we developed the YopM16 aptamer using magnetic bead-based (Systematic Evolution of Ligands by Exponential Enrichment) (SELEX). The selected YopM16 aptamer exhibited a half-maximal inhibitory concentration(IC_50_) value of 103.3 ± 2 nM and effectively inhibited the interaction between YopM and DDX3. The inhibitory effect of the aptamer on protein interaction was confirmed using a pull-down assay and colorimetric test. Given that protein–protein interaction surfaces are considered undruggable, YopM16 is a promising inhibitor with the potential to serve as a molecular tool to investigate the virulence mechanism of YopM, as well as a novel antibacterial agent upon validation of its inhibition in cellular models.

## 1. Introduction

The *Yersinia* genus includes three species that are pathogenic for humans. *Y. enterocolitica* and *Y. pseudotuberculosis* cause foodborne infections, whereas *Y. pestis* causes the plague [1]. Plague is a highly severe infectious disease in humans, particularly in its septicemic (systemic bloodstream infection) and pneumonic forms, which are associated with extremely high mortality rates ranging from approximately 30% to 100% if untreated. The plague has caused the deaths of millions of people in history and remains a re-emerging and clinically significant infectious disease, with periodic outbreaks reported in endemic regions. Recent epidemiological and One Health-based studies have emphasized that plague persists at the animal–human–environment interface, and its re-emergence is strongly influenced by ecological disruption and global environmental changes. Following the severe plague outbreak in 2017 in Madagascar, *Y. pestis* has been included by the World Health Organization (WHO) in the list of pathogens with pandemic potential [2,3]. This classification underscores the need for sustained surveillance and the development of novel therapeutic strategies against *Y. pestis* infection. As no vaccines or immunotherapeutic approaches have been approved, antibiotic treatment is the only therapeutic option for controlling the infection. However, the emergence of antibiotic-resistant *Y. pestis* strains poses a public health threat [4]. Bacteria use different mechanisms to rapidly acquire resistance to conventional antibiotics [5]. Bactericidal antibiotics cause resistance by exerting selection pressure on susceptible bacteria. Hence, targeting virulence factors [6] or protein–protein interactions [7] has been proposed as an alternative strategy to minimize the antibiotic selection pressure.

The type III secretion system (T3SS) of Gram-negative bacteria is an attractive target for developing novel antimicrobials [8]. As T3SS is not essential for growth, inhibitors of T3SS are less likely to cause selection pressure and lead to antibiotic resistance. *Yersinia* T3SS plays a significant role in virulence by manipulating host signaling pathways and immune response. The T3SS is used to inject *Yersinia* outer proteins (Yops) into the host cell, where Yop effector proteins exert immunomodulatory effects [9]. The effector proteins YopJ, YopM, YopE, YopT, YopH, YpkA, and YopK have different roles, such as inhibition of phagocytosis and suppression of immune signaling [10].

Unlike other Yops, YopM has multiple roles in immunosuppressive mechanisms [11,12]. YopM blocks the pyrin inflammasome, thereby counteracting the innate immune response triggered by YopE and YopT [13]. Inhibition of caspase-1 activity by YopM blocks the formation of mature inflammasome [14]. YopM also functions as an E3 ubiquitin ligase, which ubiquitylates NOD-like receptor (NLR)-family pyrin domain-containing 3 (NLRP3), thereby inducing NLRP3-mediated necrotic cell death [15]. The immunosuppressive function of YopM is attributed to its interaction with multiple proteins, including host cell kinases [16] and IQ motif-containing GTPase-activating protein 1 (IQGAP1) [17]. Another interaction partner of YopM is DEAD-box helicase 3 (DDX3), which shuttles YopM out of the nucleus via the calreticulin-mediated nuclear protein export (CRM1) pathway. The shuttling of YopM between the cytoplasm and nucleus is important for its immunosuppressive function. YopM maintains nuclear ribosomal S6 kinase 1 (RSK1) in a phosphorylated and active state, which in turn increases the expression of the immunosuppressive interleukin-10 [18]. Nuclear translocation of the transcription factor signal transducer and activator of transcription 3 (Stat3) is stimulated by YopM, which provides another mechanism for increasing the expression of IL-10 in macrophages. Although the mechanism of YopM-mediated nuclear translocation is unknown, it has been proposed that YopM acts as a carrier for unphosphorylated Stat3 [19].

Given the essential role of bacterial T3SS in virulence, its protein components are ideal targets for the development of therapeutic agents [20]. A small molecule inhibitor targeting YopD, a protein involved in the regulatory processes of T3SS, has been shown to inhibit the secretion of Yops by *Y. pestis* [21]. Inhibition of YscN ATPase activity by computationally selected inhibitors has impaired T3SS function, resulting in the inhibition of YopE secretion [22]. As reviewed elsewhere [23], several antibodies targeting the needle tip, translocon proteins, basal body, and effector proteins of T3SS have been developed. However, none of these have reached the market yet, due to the toxicity of small molecules or the delivery of anti-T3SS antibodies targeting the intracellular proteins. As an alternative to small-molecule drugs and antibodies, nucleic acid aptamers have emerged as potential antimicrobials [24,25]. Aptamers, first identified in 1990 [26,27], are single-stranded DNA or RNA molecules that mimic the binding properties of antibodies by folding into unique three-dimensional structures. Aptamers are selected using an in vitro method called Systematic Evolution of Ligands by Exponential Enrichment (SELEX), which enables the use of non-immunogenic targets under controlled conditions of pH, temperature, and ionic strength. Aptamers have superior properties, such as ease of chemical synthesis, low cost, small size, and low immunogenicity, making them promising therapeutic candidates. To date, the aptamers pegaptanib and avacincaptad pegol have been approved by the U.S. Food and Drug Administration (FDA), and several aptamers are under clinical trials [28,29,30].

In this study, given the essential role of *Yersinia* YopM in virulence and the lack of specific inhibitors of the YopM-DDX3 interaction, we aimed to select a DNA aptamer against YopM and evaluate its inhibitory potential to disrupt the protein–protein interaction (PPI). The development of small-molecule inhibitors that target protein–protein interactions is challenging because of the large and flat characteristics of the interaction surface, which often renders these interfaces poorly targeted by conventional small molecule drugs and thus considered “undruggable” targets in many cases. Hence, aptamers with extended surface areas for target recognition can specifically interact with proteins and effectively block protein interactions [31,32,33]. Aptamers have been proven to be successful in inhibiting the protein interactions of transcription factors [34], programmed death-ligand 1 (PD-L1) protein [35], and SARS-CoV-2 spike protein [36].

## 2. Results

### 2.1. Selection and Characterization of YopM Aptamers

**Figure 1:**
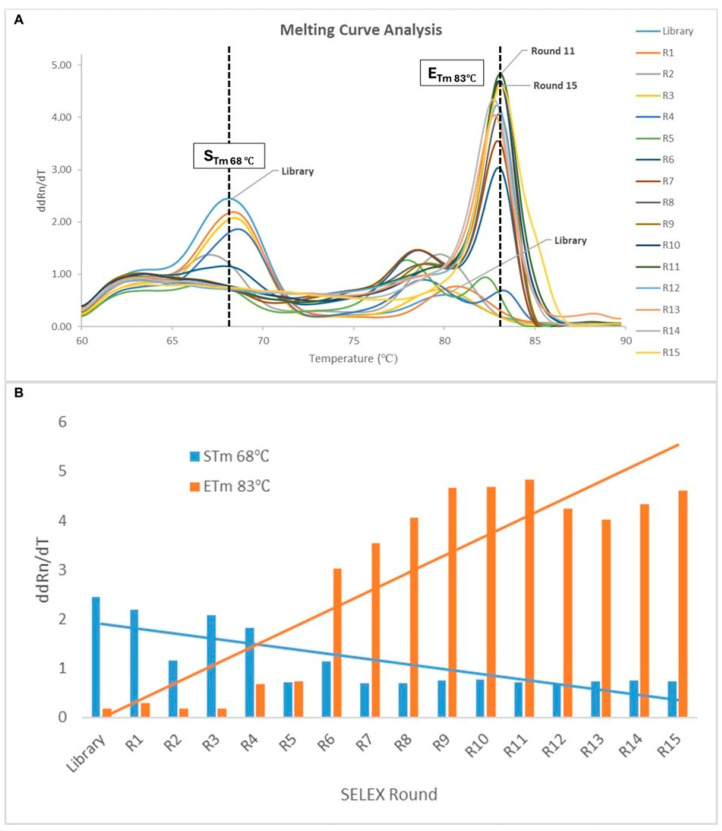
Melting curve analysis of aptamer pools from the initial library and rounds R1-R15. (**A**) Change in fluorescence divided by change in temperature (ddRn/dT) with temperature peaks at S_Tm_ (68 °C) at the beginning of SELEX and E_Tm_ (83 °C) at the end of SELEX (**B**) peak shift analyses for S_Tm_ (blue) and E_Tm_ (orange).

Aptamer selection was performed using magnetic bead-based SELEX, which allows the rapid partitioning of target-bound sequences under magnetic field. As summarized in Table S1, the amount of YopM-immobilized beads gradually decreased to increase the stringency of the binding conditions. Moreover, the washing time was increased to remove the weak binders. Real-time polymerase chain reaction (RT-PCR) melting curve analysis was performed to monitor the enrichment of sequences during the SELEX rounds. Figure 1A shows the melting curves, and Figure 1B shows the degree of shift in the melting temperature of the aptamer pool at the start (S_Tm_) and end (E_Tm_) of each SELEX round. The S_Tm_ and E_Tm_ values were also calculated for the initial library. As SELEX progressed, the average melting temperature of the library increased from 68 °C (S_Tm_) to 83 °C (E_Tm_). The increase in the E_Tm_ value indicated the enrichment of GC-rich sequences, reflecting the enrichment of aptamers with more stable structures and a higher propensity to bind YopM [37,38]. As shown in Figure 1B, the highest degree of enrichment was observed in the 11th SELEX round. However, in the 12th and 13th rounds, the E_Tm_ value decreased again, most likely because of the introduction of counter-target proteins (Table S1). Hence, the elimination of sequences binding to counter-targets was easily monitored using melting curve analysis. Following a transient decline in the E_Tm_ value, enrichment resumed in subsequent rounds, leading to the retention of higher-affinity aptamers in the pool.

**Figure 2:**
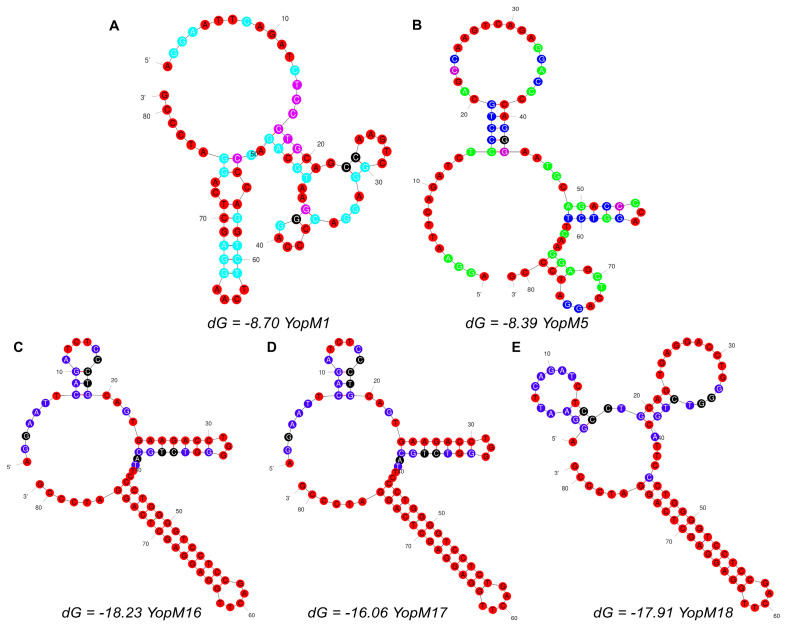
The mfold predicted secondary structures of aptamers YopM1 (**A**)**,** YopM5 (**B**), YopM16 (**C**), YopM17 (**D**), and YopM18 (**E**).

Aptamer pools from SELEX rounds 1, 11, and 15 were sequenced using next-generation sequencing (NGS) to identify aptamer sequences. The sequences were ranked based on the reads per million mapped reads (RPM) and the total read counts (Table S2). The top 15 sequences from each round were retrieved for Multiple Em for Motif Elicitatio (MEME) Suite analysis. The MEME Suite program was used to analyze the similarities between sequences (Figure S1). Five candidate aptamers with the most significant negative *p*-values were selected for further analysis. The mfold program was used to predict the secondary structures of the five candidate aptamers (Figure 2). The Gibbs free energy (ΔG) values of the predicted structures were compared (Table 1).

Isothermal titration calorimetry (ITC) measurements were performed to determine the binding affinity of the aptamers. The dissociation constant (K_d_) value for the YopM16 aptamer was estimated to be 271 nM as a lower limit, rather than a precisely defined value (Figure S2). Incomplete saturation of binding was attributed to the dimerization tendency of YopM, shifting the monomer-dimer equilibrium during the ITC experiment and altering the effective binding stoichiometry [39,40,41]. The YopM5 (K_d_ = 5.01 μM) and YopM17 (K_d_ = 9.96 μM) aptamers exhibited K_d_ values in the micromolar range (Figure S3). ITC measurements of the YopM1 and YopM18 aptamers revealed no significant binding to YopM (O. Gök, Ege University, Izmir, Türkiye, unpublished data, 2024 [42]). Among the tested aptamers, YopM16, which exhibited the highest binding affinity for YopM, was selected for further experiments. The binding specificity of YopM16 aptamer was evaluated against the proteins used as counter-targets during the SELEX rounds. ITC measurements confirmed that YopM16 did not show any non-specific binding to hemoglobin, human serum albumin *(*HSA), or DDX3 (Figure S4). These results demonstrate that the YopM16 aptamer binds specifically to YopM and does not interact with non-target proteins.

### 2.2. The Inhibitory Effect of YopM Aptamer on Protein–Protein Interaction

#### 2.2.1. Colorimetric Assay

**Figure 3:**
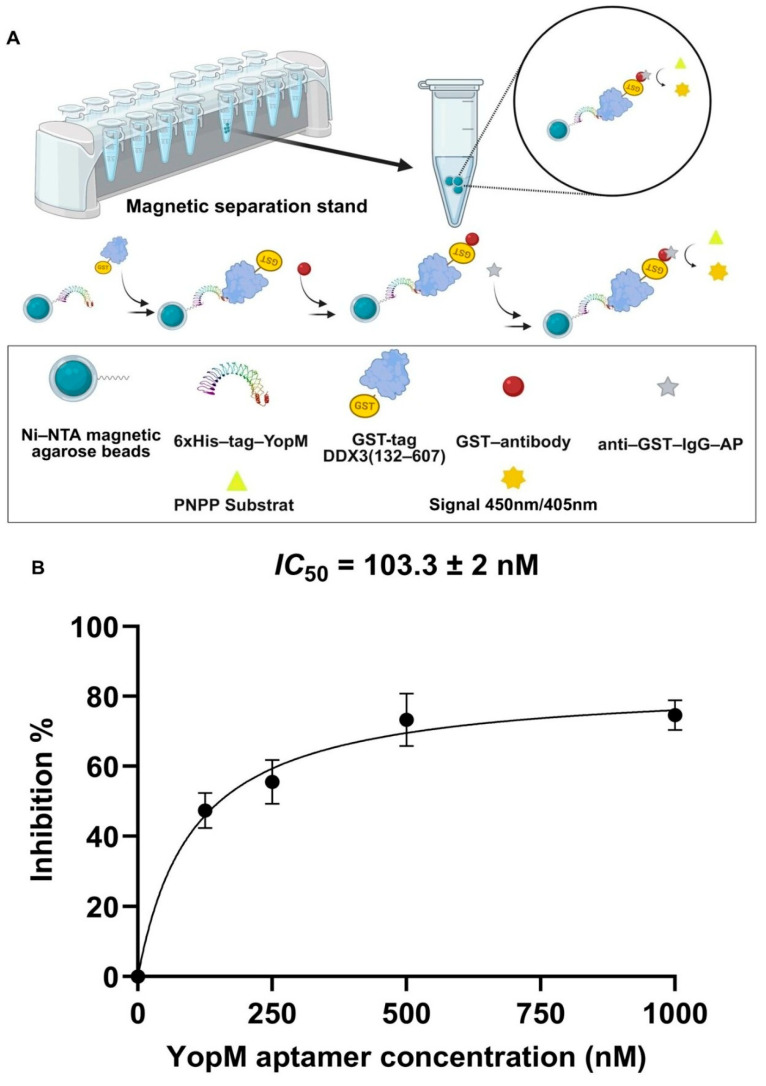
Schematic summary of the Ni-NTA magnetic agarose bead-based colorimetric assay (created with BioRender.com) (**A**). The percentage of inhibition of protein–protein interaction as a function of increasing concentrations of the YopM16 aptamer (**B**).

The effect of YopM16 aptamer on the protein–protein interaction between YopM and DDX3 was determined using a colorimetric assay used (Figure 3A). This assay involves capturing the YopM-DDX3 complex using Ni^2+^-coated magnetic beads via the 6xHis-tag of YopM, followed by the binding of anti-GST antibody to DDX3 via its GST-tag. As the GST antibody is conjugated with the alkaline phosphatase (AP), the formation of the complex is monitored by measuring the absorbance of the alkaline phosphatase reaction product. To assess the inhibitory effect of the aptamer, YopM was incubated with the YopM16 aptamer at different concentrations. Subsequently, the YopM protein-aptamer complex was incubated with DDX3, followed by a colorimetric assay. The decrease in absorbance indicates the inhibition of the protein complex by the aptamer. The percentage of inhibition was plotted against the aptamer concentration, and the IC_50_ value was calculated using GraphPad Prism 9.3.0 software. As shown in Figure 3B, the IC_50_ value of YopM16 was determined to be 103.3 ± 2 nM. The percentage of inhibition increased with increasing aptamer concentration. However, beyond a concentration of 500 nM, no significant difference in the percentage of inhibition was observed.

#### 2.2.2. Pull-Down Assay

**Figure 4:**
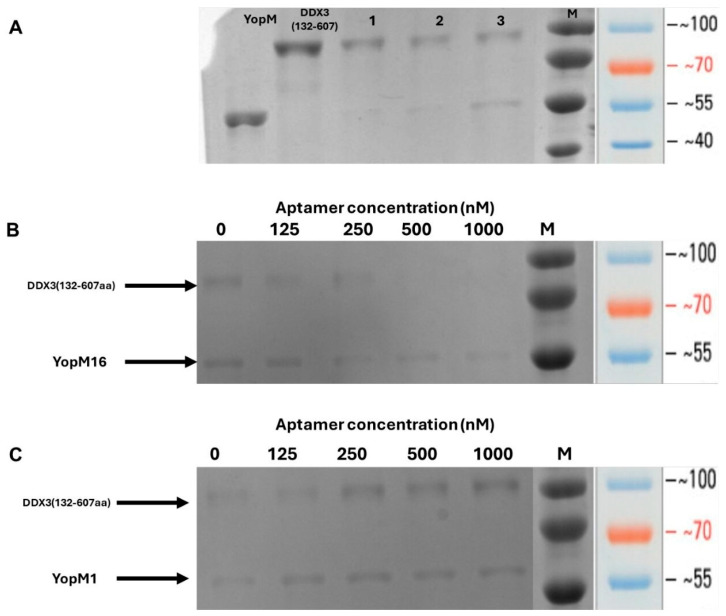
Pull-down assay results. M indicates the PageRuler™ Prestained Protein Ladder, 10–180 kDa (Waltham, MA, USA, Thermo Fisher Scientific cat. no: 26616) (**A**) Confirmation of YopM-DDX3 protein–protein interaction. Lanes 1–3 indicate YopM:DDX3 molar ratios of 0.5:1; 1:1; 2:1, respectively. (**B**) Inhibitory effect of the YopM16 aptamer on protein–protein interaction (**C**) Visualization of YopM and DDX3 protein bands in the presence of increasing concentrations of YopM1 aptamer (125, 250, 500, and 1000 nM).

As schematized in Figure S5, the pull-down assay is based on the principle of separation of protein complex using an affinity matrix, followed by SDS-PAGE analysis to reveal the proteins forming the complex. Before testing the inhibitory effect of the aptamer, the protein–protein interaction between YopM and DDX3 was confirmed using pull-down assay. For this aim, YopM and DDX3 were incubated at YopM:DDX3 molar ratios of 0.5:1, 1:1, and 2:1. As shown in Figure 4A, both YopM and DDX3 protein bands were monitored at a 2:1 molar ratio, indicating the protein–protein interaction. The inhibitory effect of the YopM16 aptamer was evaluated at a molar ratio of 2:1 (YopM:DDX3). The YopM protein band remained visible, whereas the density of DDX3 protein band gradually decreased with increasing concentrations of the YopM16 aptamer (Figure 4B). Consistent with the results of the colorimetric assay, the YopM16 aptamer effectively inhibited protein–protein interaction at concentrations of 500 nM and above. The assay was also performed in the presence of increasing concentrations of the YopM1 aptamer, which was used as a negative control because of its lack of binding, as confirmed by ITC. As shown in Figure 4C, both YopM and DDX3 protein bands remained visible, indicating no inhibitory effect of the YopM1 aptamer.

## 3. Discussion

In this study, we identified the YopM16 aptamer as an inhibitor of YopM-DDX3 protein–protein interaction. SELEX yielded several aptamer candidates, which were identified using NGS analysis. Accordingly, the aptamers YopM1, YopM5, YopM16, YopM17, and YopM18 were selected for further characterization. Sequence analysis revealed that the YopM1 and YopM5, the aptamers from the 15th SELEX round, differed by only one nucleotide (Table 1). However, as shown in Figure 2, mfold prediction yielded different secondary structures for YopM1 and YopM5. In this study, mfold was used as a frequently preferred secondary structure prediction tool, since it employs free energy minimization algorithms that incorporate temperature and ionic conditions to determine the most thermodynamically stable conformations of nucleic acid sequences [43,44]. ITC measurements showed that YopM1 did not have a significant binding to YopM, whereas YopM5 showed binding affinity with a K_d_ of 5.01 µM. The difference in the binding properties of the two aptamers shows that the unique structure of YopM5 mediates binding to YopM, albeit with a lower affinity in the micromolar range. The aptamers YopM16, YopM17, and YopM18 were obtained in the 11th SELEX round. As shown in Table 1, YopM16 differs by only one nucleotide compared to YopM17 and YopM18. However, the positions of the nucleotide changes were different. The predicted secondary structures of YopM16 and YopM17 were highly similar, whereas YopM18 exhibited structural differences (Figure 2). YopM16 showed the highest binding affinity with a K_d_ of 271 nM, whereas the K_d_ for YopM17 was calculated to be 9.96 µM. The difference between the K_d_ values of the two aptamers suggests that the single nucleotide exchange in YopM17 is critical for binding. The differences in the predicted structures of YopM16 and YopM18 also revealed the structural dependency of aptamer-target interactions. Based on the secondary structures predicted by mfold, YopM16 displayed the lowest ΔG value among the aptamers (Table 1). As low ΔG is correlated with higher thermodynamic stability, which enables the aptamer to form a stable structure and bind its target effectively [45]. Accordingly, this correlation was evident for YopM16 aptamer (5′-AGGAATTCAGATCTCCCTGCAGTGAAGACCTGGGGTCTGCATTCCCTGGGGTCCTCCGACTTGGAGGAGCTCAGGATCCCG-3′), which had both the highest affinity and the lowest ΔG. Taken together, further structural studies are needed to understand the effects of variations in aptamer structure on binding affinity and specificity. In particular, molecular dynamics simulations combined with structural bioinformatics approaches can provide insights into the structure-function relationship. Our results highlight the need for molecular dynamics studies to better understand the interaction between YopM16 and YopM, as well as to identify key nucleotide residues and secondary structural motifs responsible for specific recognition and binding to YopM.

The counter-SELEX was performed against DDX3, hemoglobin and HSA to remove the off-target binding sequences. To assess the success of counter-SELEX, the binding specificity of YopM16 was tested against hemoglobin, HSA, and DDX3 using ITC measurements. As shown in Figure S4, no significant change in DP versus time was observed, indicating no significant off-target binding of YopM16 to hemoglobin, HSA, and DDX3. A counter-SELEX step against yellow fluorescent protein *(*YFP) was also performed. The aim of using YFP as a counter-target was its future use in genetically encoded fluorescent protein-based biosensors [46,47]. Although it is beyond the scope of this study, YopM-based fluorescent biosensors can be constructed for intracellular monitoring of aptamer-mediated inhibition of DDX3–YopM. Hence, the sequences showing off-target binding to YFP were removed.

The inhibitory effect of YopM16 was assayed using both pull-down and colorimetric tests. Pull-down assays were performed using different molar ratios of YopM and DDX3. Consistent with previous findings [18], pull-down assays showed that YopM forms a 2:1 complex with DDX3. As shown in Figure 4, the pull-down assay revealed the inhibitory effect of YopM16. As the pull-down assay yielded semi-quantitative results, a colorimetric test was used to determine the IC_50_ value. The IC_50_ value for YopM16 was calculated to be 103.3 ± 2 nM. Previous studies have shown that aptamers with IC_50_ and K_d_ values in this range effectively block protein–protein interactions of transcription factors [34], as well as the adhesion of prostate cancer cells [48], entry of chikungunya virus into host cells [49], and in vivo Shiga toxin type 2 activity [50]. A previous study showed that DNA aptamer developed for integrin α6β4, which prevents prostate cancer cells from adhering to laminin-332, showed inhibition with an IC_50_ value of 149 nM and a K_d_ value of 137 nM [48]. In another study, the S4 aptamer, one of the DNA aptamers that blocks the interaction between the Stx2 B subunit and Gb3, had IC_50_ and K_d_ values of 220.4 nM and 22.07 nM, respectively [50]. Our study shows that YopM16, with a K_d_ of 271 nM and an IC_50_ of 103.3 ± 2 nM, has an inhibitory potential similar to that of previously reported aptamers.

Bacterial cells, virulence proteins and toxins have been attractive targets for aptamer selection [51,52,53,54,55,56]. The aptamers for *Yersinia* research are limited to a few studies, targeting *Y. enterocolitica* cells [57], F1 protein of *Y. pestis* [58], and *Yersinia* adhesin A (YadA) of *Y. enterocolitica* [59]. To the best of our knowledge, our study reports the first DNA aptamer for YopM protein, and also the first inhibitor of YopM-DDX3 interaction. Given that the interaction between YopM and DDX3 plays a major role in nucleocytoplasmic shuttling of YopM, the developed aptamer can be useful to interfere with the virulence mechanism of YopM.

Protein–protein interaction surfaces are considered undruggable targets. In addition, small-molecule inhibitors have disadvantages, such as toxic effects and challenging design processes. As shown in this study, the YopM16 aptamer is a promising protein–protein interaction inhibitor that can overcome the limitations of conventional inhibitors. However, further studies are needed to test the inhibitory effects of YopM16 in cellular models. Endocytic pathways play a major role in cellular internalization of aptamers [60]. Moreover, intracellular delivery of aptamers can be enhanced using lipid nanoparticle delivery systems [61] or conjugation with cell-penetrating peptides [62]. Our study lacks cell culture studies, and the intracellular delivery of YopM16 needs to be further tested. We showed that YopM16, with K_d_ and IC_50_ values at nanomolar levels, can bind YopM and inhibit its interaction with DDX3. YopM16 can be further optimized to enhance its affinity and inhibitory effect. Dimeric and multimeric aptamers have been reported to show improved target binding affinity or therapeutic activity [63,64]. Further studies should be performed to design a dimeric or multimeric aptamer that blocks a larger area on the protein interaction surface of YopM. In addition, the structural basis of inhibition should be studied. Nevertheless, the YopM16 aptamer can be used as a tool to study the virulence mechanism of YopM and facilitate the design of novel antibacterials against *Y. pestis.*

## 4. Materials and Methods

### 4.1. Purification of Recombinant YopM and DDX3

The codon-optimized gene encoding YopM from *Y. pestis* (UniProt code: P17778) was synthesized by GenScript (Piscataway, NJ, USA). YopM was expressed and purified as described in our previous study [65]. Briefly, the pET28a(+) plasmid encoding YopM was transformed into *E. coli* NEB T7 Exp. Iq RP cells (New England Biolabs, Ipswich, MA, USA). The cells were then grown at 37 °C in Luria–Bertani (LB) medium supplemented with kanamycin (50 µg/mL) and chloramphenicol (50 µg/mL). When the optical density at 600 nm (OD_600_) reached 0.4–0.6, protein expression was induced by adding 0.5 mM isopropyl β-D-1-thiogalactopyranoside (IPTG). The cells were then incubated at 25 °C with shaking at 180 rpm for 20 h. After incubation, the cells were removed from the medium by centrifugation at 4000 rpm for 30 min at 4 °C. The cell pellet was resuspended in binding buffer (100 mM KH_2_PO_4_, 500 mM NaCl, 10 mM imidazole, pH 7.8), and homogenized using an ultrasonic homogenizer (Sonics & Materials Inc., Newtown, MA, USA). Cellular debris was removed by centrifugation at 15,000 rpm for 30 min at 4 °C. The lysate was loaded onto a HisTrap™ FF column (Cytiva, Marlborough, MA, USA) connected to the ÄKTAprime plus system (Cytiva, Marlborough, MA, USA). His-tagged YopM was eluted with a buffer containing 100 mM KH_2_PO_4_, 500 mM NaCl, and 300 mM imidazole (pH 7.8).

The pHM-GWA vector encoding the DEAD-box Helicase 3 (DDX3) protein (UniProt code: O00571) was a kind gift from Prof. Jennifer A. Doudna (University of California, Berkeley, CA, USA). The gene fragment encoding amino acids 132–607 was amplified from the pHM-GWA vector using polymerase chain reaction (PCR), as described in Table S3. The amplified fragment was cloned into the pGEX 4T-1 vector in frame with glutathione S-transferase (GST) through *Bam*HI-HF and *Eco*RI-HF restriction sites [66]. The plasmid was then transformed into *E. coli* BL21 (DE3) Gold cells (Agilent Technologies, Santa Clara, CA, USA). The cells were incubated at 37 °C in LB medium containing ampicillin (150 µg/mL) and tetracycline (20 µg/mL). When the OD_600_ reached 0.4–0.6, protein expression was induced by the addition of 0.5 mM IPTG. Following incubation at 25 °C with shaking at 180 rpm for 20 h, the cells were homogenized using an ultrasonic homogenizer (Sonics & Materials Inc., Newtown, MA, USA). Following centrifugation at 15,000 rpm for 30 min at 4 °C, the cellular lysate was loaded onto a GSTrap™ HP column (Cytiva, Marlborough, MA, USA) equilibrated with the binding buffer (10 mM Na_2_HPO_4_, 2.7 mM KCl, 137 mM NaCl, 1.8 mM KH_2_PO_4_, 1 mM DTT, and 1 mM EDTA; pH 7.4). GST-tagged DDX3 (132–607) was purified using the ÄKTAprime plus system (Cytiva, Marlborough, MA, USA), and elution was achieved with 50 mM Tris buffer (pH 8.0) containing 30 mM reduced glutathione. The purity of the purified YopM and DDX3 proteins was assessed using Tricine-SDS-PAGE [67]. Protein concentration was determined using the Bradford assay [68].

### 4.2. In Vitro Selection of DNA Aptamers Against YopM

The magnetic bead-based SELEX method [69,70,71,72] was used for aptamer selection. Briefly, YopM protein was immobilized on Ni-NTA magnetic agarose beads (Jena BioScience, Jena, Thuringia, Germany; Cat. No: AC-604-5) according to the manufacturer’s protocol. First, 250 μL of beads were suspended in an equal volume of SELEX binding buffer (PBS-T; 137 mM NaCl, 2.7 mM KCl, 10 mM Na_2_HPO_4_ 1.8 mM KH_2_PO_4_, 2 mM MgCl_2_, 0.05% Tween-20, pH 7.5). The beads were washed three times with buffer. Then, 50 μg of YopM was added to 1 mL of PBS-T buffer, including 250 μL of magnetic beads. The mixture was then incubated at 25 °C for 45 min at 900 rpm. After incubation, the beads were washed three times with 250 μL PBS-T buffer. During each washing step, the supernatant was collected to measure the concentration of unbound protein. Finally, the protein-bound magnetic beads were resuspended in 250 μL PBS-T buffer. The YopM-immobilized magnetic beads were stored at 4 °C until further use.

**Figure 5:**
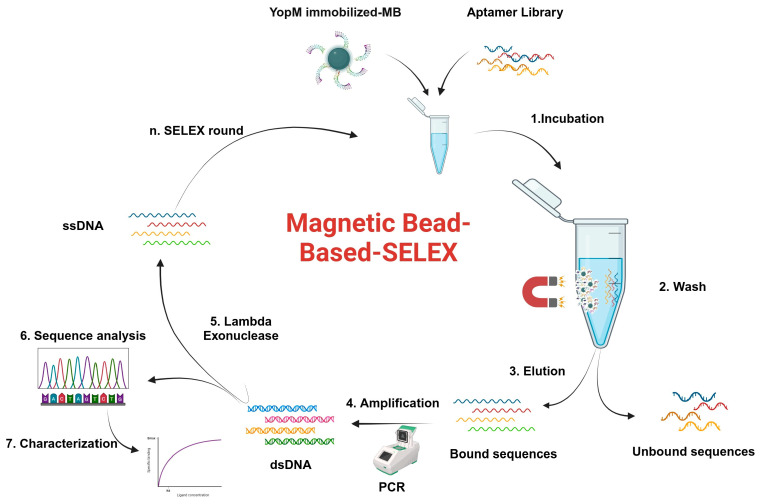
Schematic representation of the magnetic bead-based SELEX method. YopM protein was immobilized onto Ni-NTA magnetic beads and incubated with the ssDNA library. Unbound sequences were removed by washing. The bound sequences were eluted and amplified by PCR, followed by lambda exonuclease digestion. The ssDNA pool was used in the next round of SELEX. The ssDNA pool obtained from the final round was subjected to sequencing analysis (Created with BioRender).

The single-stranded DNA (ssDNA) library with a 42 nucleotide-randomized region (5′-CACGACGCAAGGACCACAGG-N42-CAGCACGACACCGCAGAGGCA-3′) was synthesized by ELLA Biotech (Fürstenfeldbruck, Bavaria, Germany). The binding buffer consisted of 10 mM Na_2_HPO_4_, 2.7 mM KCl, 137 mM NaCl, 1.8 mM KH_2_PO_4_, 0.05% Tween-20, and 2 mM MgCl_2_ (pH 7.5). As schematized in Figure 5, SELEX consists of iterative rounds of binding, elution of binders, and PCR amplification. As summarized in Table S1, the stringency was increased in each round by decreasing the amount of YopM and increasing wash time. Before each round of SELEX, the ssDNA pool was incubated at 95 °C, 4 °C, and then at 25 °C for 5 min. In the first round, negative-SELEX was performed to remove the sequences that bound to the naked Ni-NTA magnetic beads. For this aim, the ssDNA library (300 pmol) was incubated with pre-washed magnetic beads. The magnetic beads were removed using a magnetic separation rack (New England Biolabs, Ipswich, MA, USA), and the unbound ssDNA pool in the supernatant was incubated with magnetic beads immobilized with 200 pmol of YopM. After incubation, the beads were washed with the binding buffer. The washed beads were then incubated in the binding buffer at 80 °C for 10 min to elute the sequences bound to the YopM protein. The eluted sequences were amplified by PCR (Table S4) to generate double-stranded DNA (dsDNA). The ssDNA was then obtained by lambda exonuclease digestion [73]. The ssDNA pool was purified using the NucleoSpin Gel and PCR Cleanup kit (Macherey-Nagel, Düren, North Rhine-Westphalia, Germany) according to the manufacturer’s instructions. A total of 15 SELEX rounds were performed, and negative-SELEX was applied in all rounds except round 14. Counter-SELEX was applied to eliminate the aptamers binding to off-target proteins, including DDX3, hemoglobin, human serum albumin (HSA), and yellow fluorescent protein (YFP). Counter-target proteins were immobilized on magnetic beads. The DDX3 protein (UniProt code: O00571) was immobilized on GST-magnetic agarose beads (Jena BioScience, Germany; Cat. No: AC-605-5) following the manufacturer’s protocol. A total of 50 μg of protein was bound to 125 μL of magnetic beads. Immobilization of HSA (Sigma-Aldrich, St. Louis, MI, USA; Cat. No: A1887-1G), hemoglobin (Sigma-Aldrich, St. Louis, MI, USA; Cat. No: H-2625), and YFP (heterologously expressed and purified in our previous unpublished study) on Dynabeads™ M-280 Tosylactivated magnetic beads (Invitrogen Life Technologies AS, Oslo, Norway; Cat. No: 14203) was performed using the manufacturer’s recommended protocol for covalent immobilization. For these proteins, 100 μg of protein was bound to 165 μL tosyl-activated magnetic beads. For counter-SELEX, the ssDNA pool was first incubated with counter protein-immobilized magnetic beads. After incubation, the magnetic beads with sequences that remained bound to the counter protein were removed using a magnet. The supernatant, which included the unbound sequences, was incubated with YopM in the next SELEX round.

### 4.3. Monitoring the Enrichment of Sequences and Next-Generation Sequencing

Melting curve analysis was performed to monitor the enrichment of the ssDNA pool obtained from each SELEX round [37,74]. For this aim, ~10 ng of PCR product from each SELEX round was mixed with iTaq SYBR Green reagent (Bio-Rad Laboratories, Hercules, CA, USA) in a total volume of 20 μL, according to the kit instructions (Table S5). For the melting curve analysis, the post-amplification temperature was gradually increased from 40 °C to 95 °C at 0.7 °C/min using a real-time PCR system (Roche, Mannheim, Baden-Württemberg, Germany). The change in fluorescence intensity was continuously monitored, and melting peaks were analysed using the instrument’s software.

The ssDNA pools identified to be enriched according to the melting curve analysis were amplified using primers with barcode sequences (Table S6) [75]. After PCR amplification, the DNA samples were purified using the PCR Clean-up kit (Macherey-Nagel, Düren, North Rhine-Westphalia, Germany). Next-generation sequencing (NGS) and bioinformatic analysis were performed by Galseq (Milan, Lombardy, Italy).

The MEME-suite web server was used for similarity analysis of the sequences identified by NGS [76]. Candidate aptamers with the highest enrichment and sequence similarity were identified using the MEME-suite analysis. The mfold web server was used to predict the secondary motifs of aptamers [77]. The conditions for secondary structure predictions were set as 137 mM Na^+^, 2 mM Mg^2+^, and 25 °C.

### 4.4. Isothermal Titration Calorimetry (ITC) Measurements

Isothermal titration calorimetry (ITC) [78,79,80,81] was used to assess the binding affinity (K_d_) of the aptamers. Measurements were performed using MicroCal PEAQ-ITC (Microcal-Malvern Panalytical, Malvern, Worcestershire, UK). ITC experiments were performed at 25 °C with a reference power of 10 μcal/s, stirring speed of 650 rpm, and spacing time of 150 s. The measuring cell volume was 200 μL and the syringe volume was 40 μL. The measurements were performed in 19 injection mode in the binding buffer used in the SELEX rounds. The protein concentration was adjusted to 5–20 μM, and titration was performed using the candidate aptamers (75–200 μM). To test the specificity of the aptamer, ITC measurements were repeated for the non-target proteins DDX3, hemoglobin and human serum albumin (HSA). Titration curves were generated using instrument’s software. The data were analyzed using the PEAQ-ITC analysis software, version 1.41 (MicroCal–Malvern Panalytical) with the one-binding-site model.

### 4.5. Analysis of Inhibition of the YopM–DDX3 Interaction by the Selected Aptamer

#### 4.5.1. Ni-NTA Magnetic Agarose Bead-Based Colorimetric Assay

To determine the inhibitory effect of the aptamer, a magnetic bead-based colorimetric assay developed by Dawei et al. [82] was performed. Briefly, Ni-NTA beads were incubated with His-tagged YopM (0.1 mg/mL) at 37 °C for 30 min with shaking at 950 rpm. After incubation, the beads were washed with the binding buffer to remove unbound YopM. The aptamer at various concentrations (125, 250, 500, and 1000 nM) was added to the YopM-immobilized magnetic beads and incubated at 37 °C for 30 min with shaking at 950 rpm. Following the addition of DDX3 (132–607), the mixture was incubated at 37 °C and 950 rpm for 45 min. The beads were washed with buffer and 100 μL of anti-GST antibody (diluted 1:4000) was added. Subsequently, 100 μL of anti-GST-IgG-antibody-alkaline phosphatase (diluted 1:5000) was added. The beads were resuspended in 100 µL of buffer and transferred to a 96-well microplate. For the colorimetric assay, 100 μL of substrate solution including 2 mg/mL p-nitrophenyl phosphate (p-NPP) dissolved in 200 mM Tris-base,10 mM MgCl_2_, 200 mM NaCl (pH 8.5), was added and incubated at 37 °C for 30 min. Absorbance was measured at 405 nm. GraphPad Prism 9.3.0 software was used to calculate the IC_50_ value. Nonlinear regression analysis was performed using the “Dose–Response–Inhibition” equation family with the “log(inhibitor) vs. response–Variable slope (four parameters)” model.

#### 4.5.2. Pull-Down Assay

Pull-down assay [82,83,84] was performed to confirm the protein–protein interaction between YopM and DDX3, as well as to investigate the inhibitory effect of the aptamer. His-tagged YopM (0.1 mg/mL) was immobilized on Ni-NTA magnetic beads and incubated with the aptamer at 37 °C and 300 rpm for 30 min. The beads were then washed with the binding buffer and GST-tagged DDX3 (132–607) (0.14 mg/mL) was added. Following incubation at 37 °C for 30 min with shaking at 300 rpm, the beads were washed with the binding buffer to remove unbound DDX3. An aliquot of the mixture was loaded onto a 12% SDS–PAGE gel and the protein bands were identified. The same steps were repeated in the absence of the aptamer.

## Figures and Tables

**Table 1:** Candidate aptamers and ΔG values for the predicted secondary structures (constant primer binding sites are underlined, and nucleotide differences are shown in red and bold). The nucleotide difference between YopM1 and YopM5 is indicated in red. The nucleotide differences between YopM17 and YopM18 compared to YopM16 are indicated in red.

Aptamer	Aptamer Sequence (5′ → 3′)	ΔG (kJ/mol)
**YopM1**	AGGAATTCAGATCTCCCTGCAGCCAAGTC**G**GAGGACCCCAGGAATGCAGACCCCAGGTCTTCAAGGAGCTCAGGATCCCG	−8.70
**YopM5**	AGGAATTCAGATCTCCCTGCAGCCAAGTC**A**GAGGACCCCAGGGAATGCAGACCCCAGGTCTTCAAGGAGCTCAGGATCCCG	−8.39
**YopM16**	AGGAATTCAGATCTCCCTGCAGTGAAGACCTGGGGTCTGCATTCCCTGGGGTCCTCCGACTTGGAGGAGCTCAGGATCCCG	−18.23
**YopM17**	AGGAATTCAGATCTCCCTGCAGTGAAGACCTGGGGTCTGCATTCCCTGGGGTCCTC**T**GACTTGGAGGAGCTCAGGATCCCG	−16.06
**YopM18**	AGGAATTCAGATCTCCCTGCAGTGA**G**GACCTGGGGTCTGCATTCCCTGGGGTCCTCCGACTTGGAGGAGCTCAGGATCCCG	−9.75

## Data Availability

The raw data supporting the conclusions of this article will be made available by the authors on request.

## Supplementary Materials

The following supporting information can be downloaded at: https://www.mdpi.com/article/10.3390/ijms27094038/s1.
