# Supplementary material for: Targeting Undruggable Protein Interactions with DNA Aptamers: Inhibition of the Interaction Between Yersinia Outer Protein M and Human DEAD-Box Helicase 3
Source: Int J Mol Sci. 2026 Apr 30;27(9):4038. doi: 10.3390/ijms27094038 (PMC13163683; doi:10.3390/ijms27094038)
Supplement: Supplementary file 1 [file ijms-27-04038-s001.zip › ijms-4282347-supplementary.pdf]

Table S1. Binding and washing conditions in each SELEX round

SELEX round	YopM-MB* (pmol)	Incubation time	Wash time	Counter molecule
**1**	200	30 min	3x1 min	-
**2**	100	30 min	3x1 min	-
**3**	100	30 min	2x1 min<br>1x3 min	-
**4**	50	30 min	2x1 min<br>1x3 min	-
**5**	25	30 min	3x3 min	-
**6**	10	30 min	1x3 min<br>2x5 min	6.50 pmol<br>hemoglobin-MB
**7**	10	30 min	3x5 min	-
**8**	10	30 min	3x5 min	10 pmol hemoglobin-MB
**9**	5	30 min	3x5 min	-
**10**	5	30 min	3x5 min	10 pmol HSA-MB
**11**	2.2	30 min	4x5 min	0.1 mg/mL<br>salmon sperm DNA
**12**	2.2	30 min	3x5 min	10 pmol YFP-MB
**13**	2.2	30 min	3x5 min	10 pmol<br>DDX3 (132-607)-MB
**14**	2.2	30 min	3x5 min	-
**15**	2.2	30 min	3x5 min	10 pmol<br>DDX3 (132-607)-MB

*MB: Magnetic beads

Table S2. Sequences obtained based on NGS reads and RPM values

Round	Rank	Reads	RPM	Sequence
**R15**	1	320455	671368.65	CCAAGTCGGAGGACCCCAGGGAATGCAGACCCCAGGTCTTCA
	2	27699	58030.74	CGGCTGATAGAACATCACAATACTGGACCTTGGCGGGCACGT
	3	23878	50025.56	CATGTCGCTATCGGACACCCATCAAAGGGCCATGAGTGGTGA
	4	19390	40622.98	TCACCACTCATGGCCCCTTGGTGGGTGTCCGATAGCGACATG
	5	3735	7825.01	CCAAGTCAGAGGACCCCAGGGAATGCAGACCCCAGGTCTTCA
	6	3447	7221.63	TGAGGACCTGGGGTCTGCATTCCCTGGGGTCCTCCGACTTGG
	7	3373	7066.59	CCAAGTCGGAGGACCCCAGGGAATGCAGACCCCAGGTCTTCG
	8	2973	6228.58	TGGAGACCTGGGGTCTGCATTCCCTGGGGTCCTCCGACTTGG
	9	2910	6096.59	CCAAGTTGGAGGACCCCAGGGAATGCAGACCCCAGGTCTTCA
	10	2156	4516.93	TGAAGACCTGGGGTCTGCATTCCCTGGGGTCCTCCGACTTAG
	11	2138	4479.21	CCAAGTCGGGGGACCCCAGGGAATGCAGACCCCAGGTCTTCA

	12	1874	3926.12	CCAAATCGGAGGACCCCAGGGAATGCAGACCCCAGGTCTTCA
	13	1826	3825.56	CCAAGTCGGAGGACCCCAGGGAATGCAGACCCCAGGTCTTTA
	14	1770	3708.24	TGAAGACCTGGGGTCTGCATTCCCTGGGGTCTTCCGACTTGG
	15	1749	3664.24	CCAAGTCGAAGGACCCCAGGGAATGCAGACCCCAGGTCTTCA
**R11**	16	1481344	824284.54	TGAAGACCTGGGGTCTGCATTCCCTGGGGTCCTCCGACTTGG
	17	12907	7182.02	TGAAGACCTGGGGTCTGCATTCCCTGGGGTCCTCTGACTTGG
	18	9641	5364.67	TGAGGACCTGGGGTCTGCATTCCCTGGGGTCCTCCGACTTGG
	19	9362	5209.43	TGGAGACCTGGGGTCTGCATTCCCTGGGGTCCTCCGACTTGG
	20	9072	5048.06	CCAAGTCGGAGGACCCCAGGGAATGCAGACCCCAGGTCTTCG
	21	8968	4990.19	CCAAGTTGGAGGACCCCAGGGAATGCAGACCCCAGGTCTTCA
	22	7168	3988.59	CCAAGTCGGGGGACCCCAGGGAATGCAGACCCCAGGTCTTCA
	23	6748	3754.88	TGAAGACCTGGGGTCTGCATTCCCTGGGGTCCTTCGACTTGG
	24	6689	3722.05	TCAAGTCGGAGGACCCCAGGGAATGCAGACCCCAGGTCTTCA
	25	6475	3602.97	TGAAGACCTGGGGTCTGCATTCCCTGGGGTCCTCCGATTTGG
	26	6410	3566.80	CTAAGTCGGAGGACCCCAGGGAATGCAGACCCCAGGTCTTCA
	27	6080	3383.18	TAAAGACCTGGGGTCTGCATTCCCTGGGGTCCTCCGACTTGG
	28	4742	2638.65	CCAAGTCGGAAGACCCCAGGGAATGCAGACCCCAGGTCTTCA
	29	3681	2048.27	TGAAGACCTGGGGTCTGCATTCCCTGGGGTCCTCCGGCTTGG
	30	3492	1943.10	CCGAGTCGGAGGACCCCAGGGAATGCAGACCCCAGGTCTTCA
**R1**	31	1248	7192.05	CCAAGTCGGAGGACCCCAGGGAATGCAGACCCCAGGTCTTCA
	32	95	547.47	TCACCACTCATGGCCCTTTGATGGGTGTCCGATAGCGACATG
	33	70	403.40	CATGTCGCTATCGGACACCCACCAAGGGGCCATGAGTGGTGA
	34	67	386.12	ACGTGCCCGCCAAGGTCCAGTATTGTGATGTTCTATCAGCCG
	35	15	86.44	CCAAGTCGGAGGACCCCAGGGAATGCAGACCCCAGGTCCTCA
	36	15	86.45	CCAAGTCGGAGGACCCCAGGGAATGCAGACCCCAGGTCTTCG
	37	15	86.45	CCAAGTCGGAGGACCCCAGGGAATGCAGACCCCAGGTCTCCA
	38	14	80.68	CCAAGTCAGAGGACCCCAGGGAATGCAGACCCCAGGTCTTCA
	39	12	69.16	TGAAGCCCTGGGGTCTGCATTCCCTGGGGTCCTCCGACTTGG
	40	11	63.39	CCAAGTCGGAGGCCCCCAGGGAATGCAGACCCCAGGTCTTCA
	41	11	63.39	CCAAGTCGAAGGACCCCAGGGAATGCAGACCCCAGGTCTTCA
	42	11	63.39	CCGAGTCGGAGGACCCCAGGGAATGCAGACCCCAGGTCTTCA
	43	10	57.63	CCAAGTTGGAGGACCCCAGGGAATGCAGACCCCAGGTCTTCA
	44	8	46.11	CCAAGTCGGAGGACCCCAGGGAATGCAGCCCCCAGGTCTTCA

	45	8	46.11	CCGTGCCCGCCAAGGTCCAGTATTGTGATGTTCTATCAGCCG

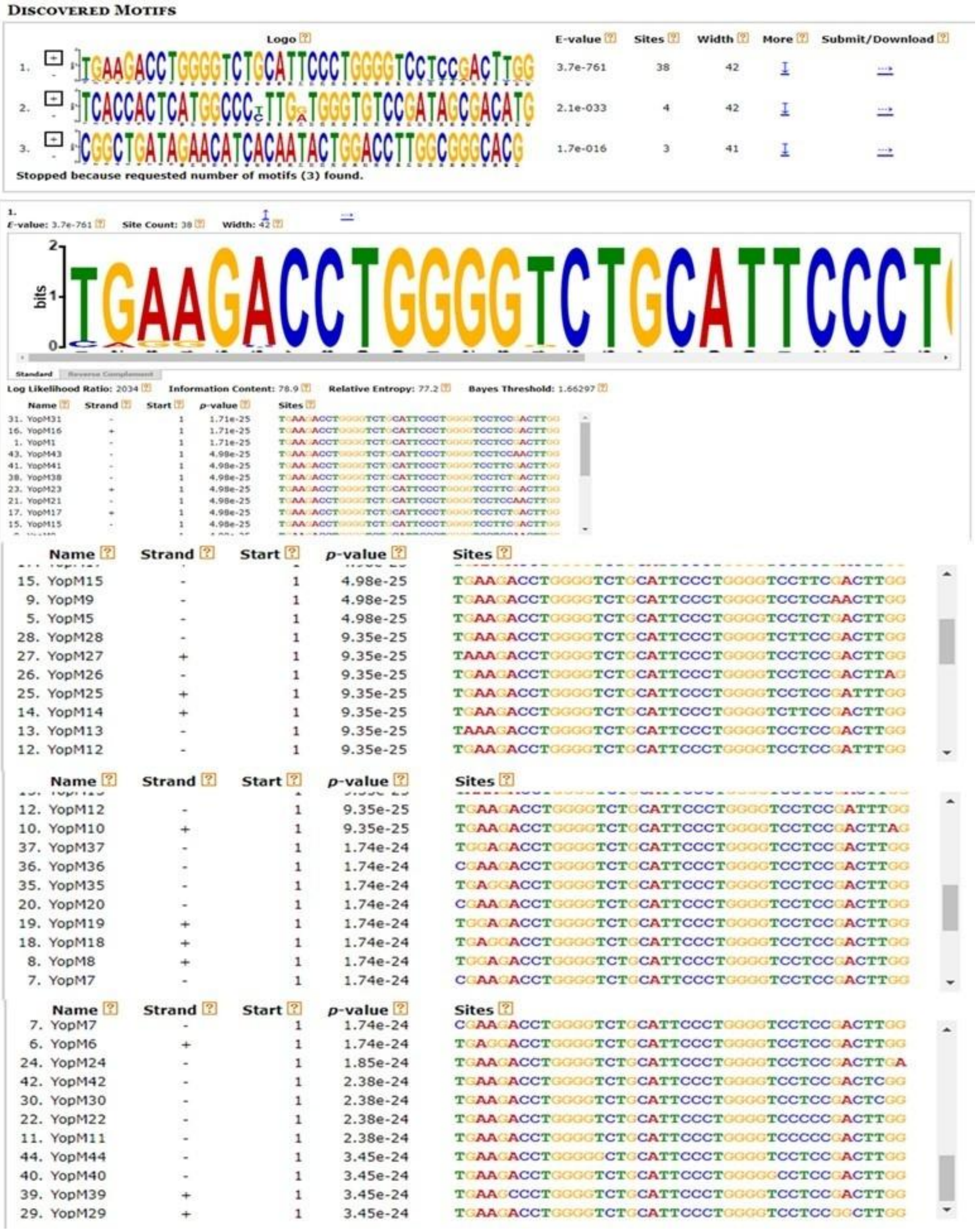

Figure S1. MEME Suit analysis for the identification of sequence similarity.

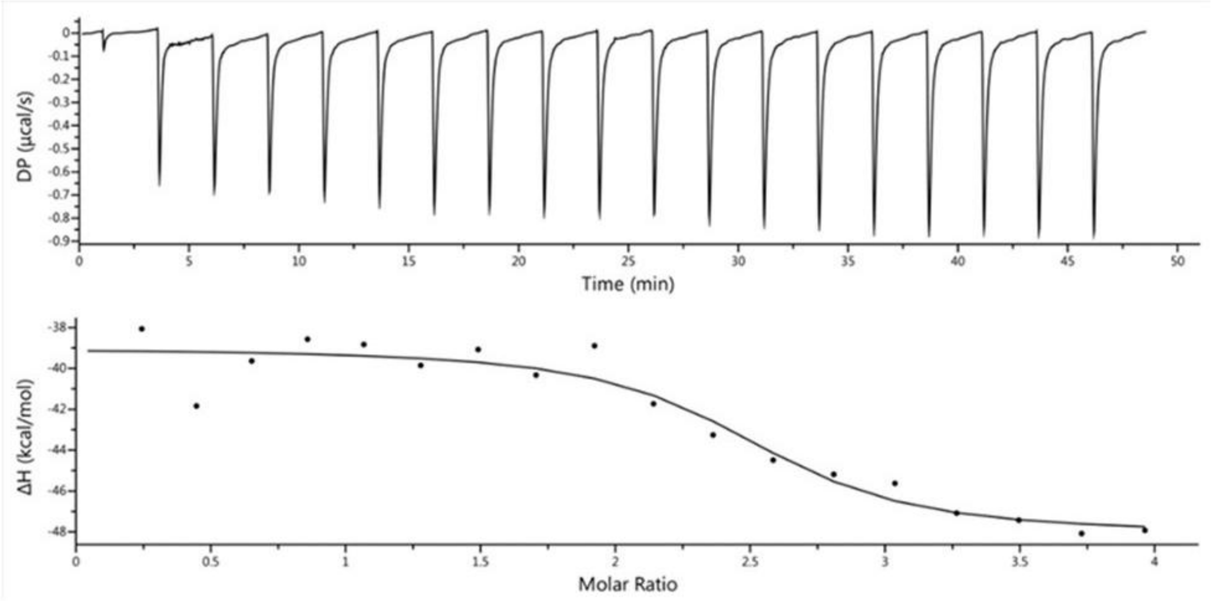

Figure S2. ITC results for YopM16 aptamer. (**A)** Raw ITC data; differential power (DP) (µcal/s) versus time (min) **(B)** enthalpy (ΔH, kcal/mol)) change plotted versus YopM:aptamer molar ratio (7.5 µM YopM was titrated with 150 µM YopM16 aptamer at 25 °C).

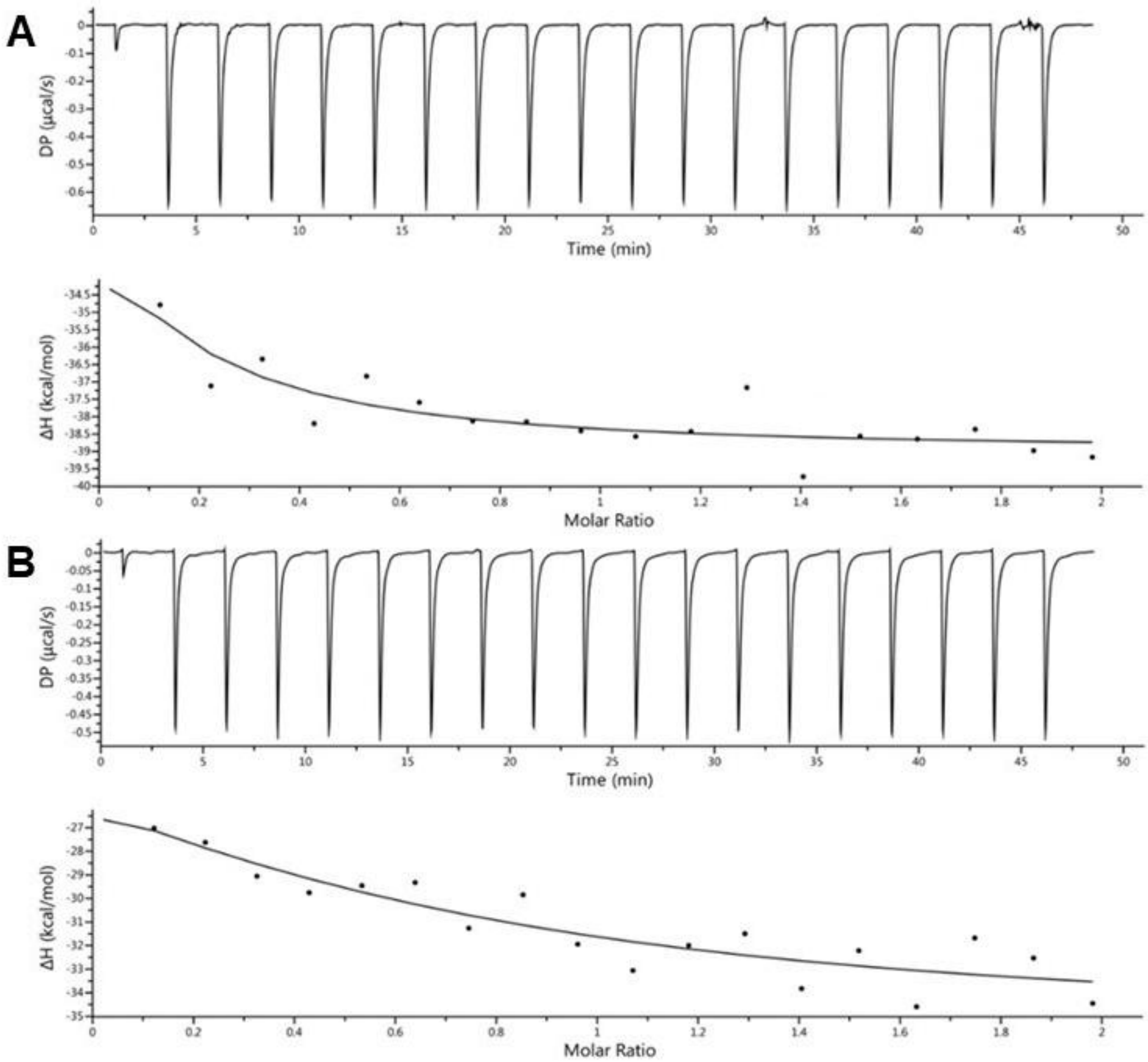

Figure S3. ITC results. (A) 10 µM YopM protein – 100 µM YopM5 aptamer, **(B)** 10 µM YopM protein – 100 µM YopM17 aptamer.

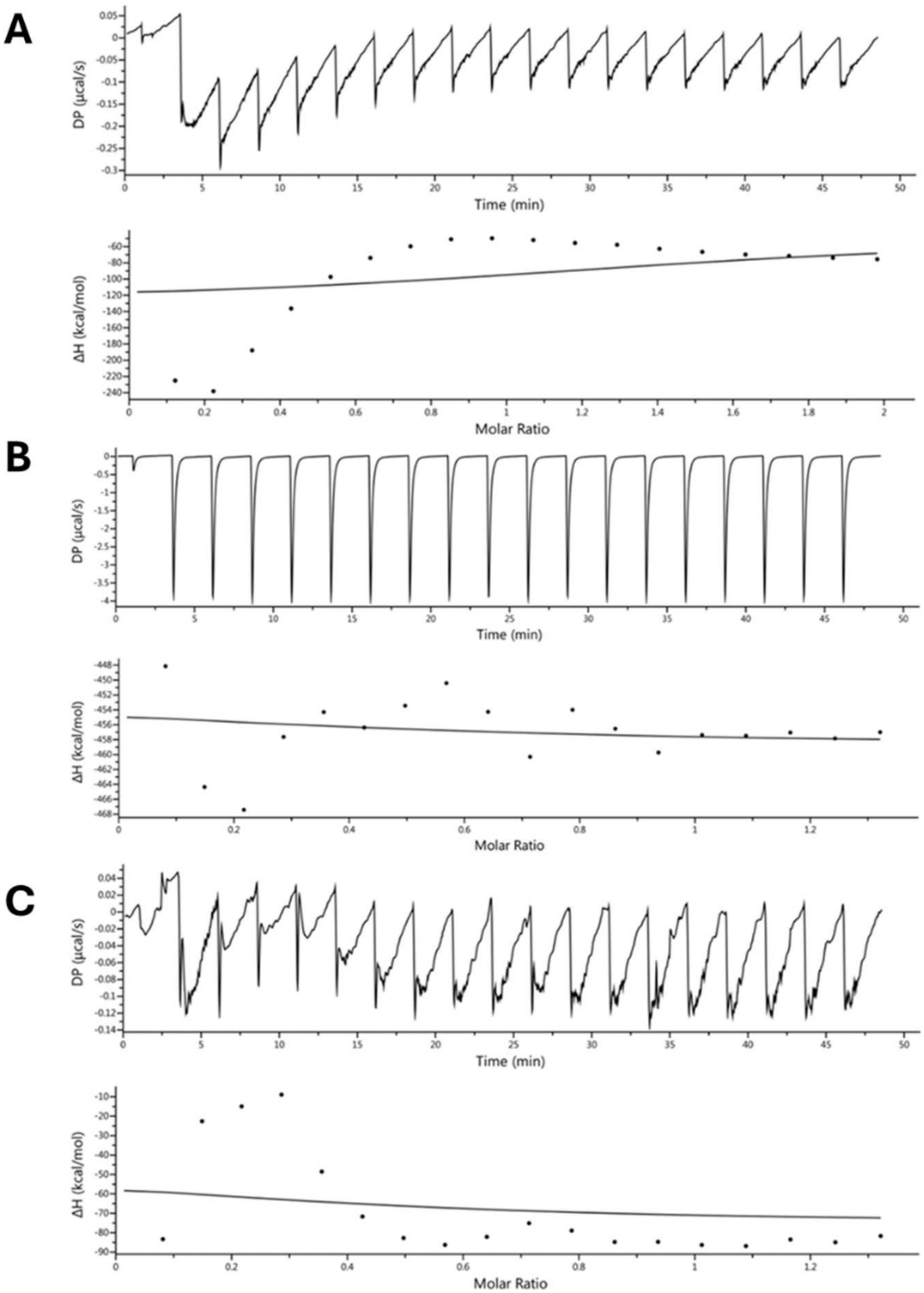

Figure S4. ITC results. **(A)** 7.5 µM Hemoglobin – 50 µM YopM16 aptamer, **(B)** 7.5 µM HSA – 50 µM YopM16 aptamer, **(C)** 7.5 µM DDX3 protein– 50 µM YopM16 aptamer.

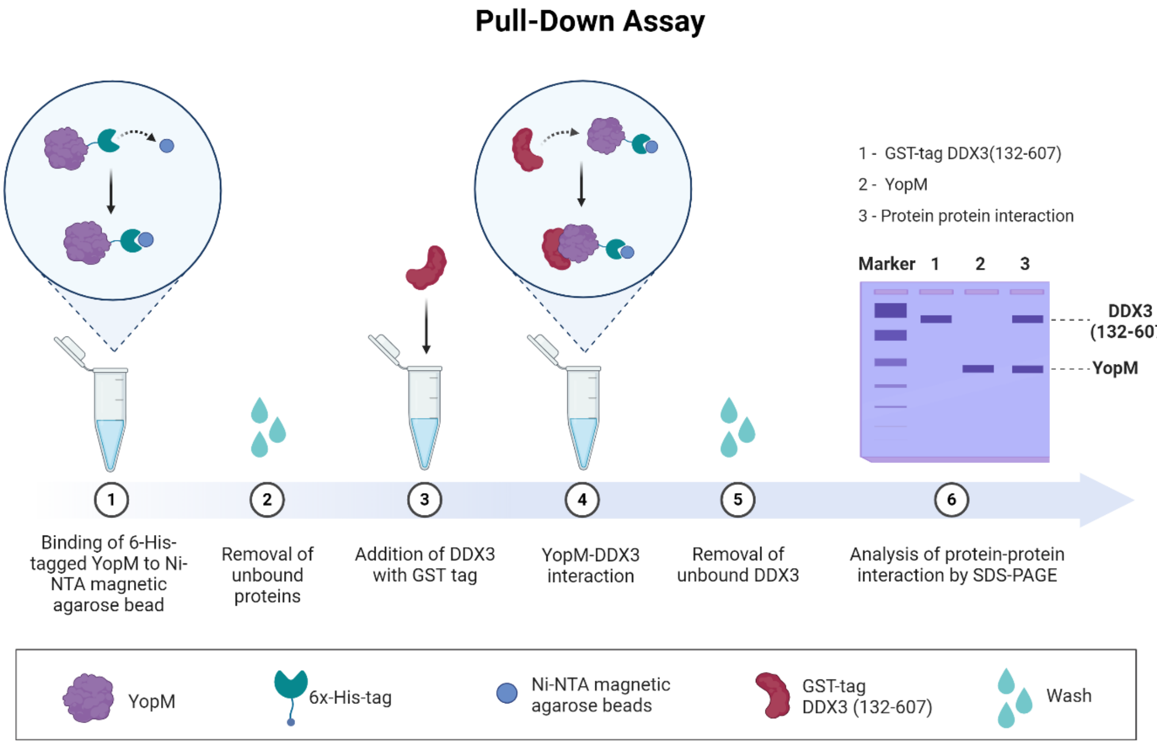

Figure S5. Schematic representation of pull-down test. Pull-down assay is based on monitoring interaction between two proteins on SDS-PAGE. If two proteins interact, both protein bands appear in the same well on the gel after electrophoresis. In the absence of interaction, one of the bands does not appear. In experiments involving aptamers, one of the protein bands disappears due to the blocking of the protein-protein interaction (created with BioRender).

Table S3. PCR components and thermal cycling protocol for amplification of the DDX3 (132-607) gene fragment

PCR mix		
**Component**	**Final concentration**	
10X Taq DNA polymerase buffer	1X	
$MgCl_2$ (25mM)	1.5 mM	
dNTPs (each 10 mM)	0.2 µM	
DDX3(132-607)-fw-*Bam*HI-HF (100 µM)	0.5 µM	
DDX3(132-607)-rv-*Eco*RI-HF (100 µM)	0.5 µM	
Template DNA	~1.0 ng/100 µL	
Taq DNA polymerase (5 U/µL)	1.25 U/µL	
H_2O	-	
**PCR protocol**		
**Step**	**Temperature**	**Time**
1. Initial denaturation	95 °C	5 min
2. Denaturation	95 °C	30 s
3. Annealing	66 °C	30 s
4. Extension	72 °C	2 min
5. Final extension	72 °C	10 min
Steps 2, 3 and 4 were repeated 30 times.		

Table S4. PCR conditions applied during SELEX rounds

PCR mix		
**Component**	**Final concentration**	
Taq DNA polymerase buffer (10X)	1X	
$MgCl_2$ (25 mM)	1.5 μM	
dNTPs (each 10 mM)	0.2 μM	
N_{42} forward primer (100 μM)	0.5 μM	
N_{42} reverse primer (100 μM)	0.5 μM	
DTT (100 μM)	2 μM	
ssDNA	*	
Taq DNA polymerase (5 U/ μL)	2.5 U	
H_2O		
*The ssDNA concentration differs in each SELEX round.		
**PCR protocol**		
**Step**	**Temperature**	**Time**
1.Initial denaturation	95 °C	3 min
2.Denaturation	95 °C	30 s
3.Annealing	64 °C	30 s
4.Extension	72 °C	30 s
5.Final extension	72 °C	2 min
Steps 2, 3 and 4 were repeated 9-21 times.		

Table S5. Real time-PCR mix for melting temperature analysis

Component	Final concentration
N_{42} forward primer (10 μM)	0.5 μM
N_{42} reverse primer (10 μM)	0.5 μM
dsDNA	*
ITaq SYBR Green Super Mix	10 μL
H_2O	-
*The dsDNA concentration differs in each SELEX round.	

Table S6. PCR primers used for NGS sample preparation

**F2**	5’-CGATGTAGGAATTCAGATCTCCCTGCAG-3’
**R2**	5'-ACATCGCGGGATCCTGAGCTCCTCGAG-3'
**F3**	5'-TTAGGCAGGAATTCAGATCTCCCTGCAG-3'
**R3**	5'-GCCTAACGGGATCCTGAGCTCCTCGAG-3'
**F4**	5'-TGCCCAAGGAATTCAGATCTCCCTGCAG-3'
**R4**	5'-TGGTCACGGGATCCTGACCTCCTCGAG-3'
F: forward primer, R: reverse primer	
